# The SUMO-targeted ubiquitin ligase RNF4 regulates localization and function of the HTLV-1 oncoprotein Tax

**DOI:** 10.1186/1742-4690-8-S1-A126

**Published:** 2011-06-06

**Authors:** Kimberly A Fryrear, Oliver Kerscher, Oliver J Semmes

**Affiliations:** 1 Department of Microbiology and Molecular Cell Biology, Eastern Virginia Medical School, Norfolk, Virginia, 23508, USA; 2 Department of Biology, College of William and Mary, Williamsburg, Virginia, 23187, USA; 3 The Leroy T Canoles Jr. Cancer Research Center, Eastern Virginia Medical School, Norfolk, Virginia, 23508, USA

## 

HTLV-1 is the etiologic agent for ATL. The virally encoded regulatory protein Tax is critical to the oncogenic process and a variety of biological activities are attributed to the protein. Tax localizes to the nucleus where it has been reported to impair the cellular DNA damage response and activate viral and cellular gene transcription. Tax also exhibits nucleocytoplasmic shuttling and in response to DNA damage translocates to the cytoplasm where it initiates activation of anti-apoptotic NFκB signaling. Thus, subcellular compartmentalization drives Tax function and likely plays an important role in HTLV-1 biology and host disease development. Although it seems clear that post-translational modification of Tax by ubiquitin/SUMO dictates subcellular localization, it is not clear how this process is regulated. We present evidence that RNF4, a SUMO-targeted ubiquitin ligase (STUbL), mediates nuclear-cytoplasmic translocation of Tax. Over-expression of RNF4 but not an RNF4 RING mutant resulted in a dramatic cytoplasmic enrichment of Tax. Concomitant with the RNF4-induced cytoplasmic accumulation of Tax was an increase in “cytoplasmic” (NFkB activation) and decrease in “nuclear” (HTLV-LTR activation) Tax functions. We demonstrated that RNF4 binds adjacent to the putative Tax ubiquitin/SUMO modification sites K280/K284 suggesting that Tax may be a substrate for RNF4. Indeed, we show that RNF4, but not RNF4 RING mutant, ubiquitylates Tax both in vitro and in vivo. Finally, depletion of RNF4 by RNAi prevents the nuclear-cytoplasmic translocation of Tax following DNA damage. These results provide important new insights into STUbL mediated pathways that regulate the functional dynamics of viral oncogenes.

